# *Ptf1a* inactivation in adult pancreatic acinar cells causes apoptosis through activation of the endoplasmic reticulum stress pathway

**DOI:** 10.1038/s41598-018-34093-4

**Published:** 2018-10-25

**Authors:** Morito Sakikubo, Kenichiro Furuyama, Masashi Horiguchi, Shinichi Hosokawa, Yoshiki Aoyama, Kunihiko Tsuboi, Toshihiko Goto, Koji Hirata, Toshihiko Masui, Yuval Dor, Tomoyuki Fujiyama, Mikio Hoshino, Shinji Uemoto, Yoshiya Kawaguchi

**Affiliations:** 1 Department of Hepato-Biliary-Pancreatic Surgery and Transplantation, Kyoto University Graduate School of Medicine, Kyoto, Japan; 2 Department of Clinical Application, Center for iPS cell Research and Application, Kyoto, Japan; 3 Department of Developmental Biology and Cancer Research, The Institute for Medical Research Israel-Canada, Hebrew University-Hadassah Medical School, Jerusalem, Israel; 4 Department of Biochemistry and Cellular Biology, National Institute of Neuroscience, NCNP, Tokyo, Japan; 5 International Institute for Integrative Sleep Medicine (WPI-IIIS), University of Tsukuba, Tsukuba, Japan

## Abstract

Pancreas transcription factor 1 subunit alpha (PTF1A) is one of the key regulators in pancreatogenesis. In adults, it transcribes digestive enzymes, but its other functions remain largely unknown. Recent conditional knockout studies using *Ptf1a*^*CreER*/*floxed*^ heterozygous mouse models have found PTF1A contributes to the identity maintenance of acinar cells and prevents tumorigenesis caused by the oncogenic gene *Kras*. However, *Ptf1a* heterozygote is known to behave differently from homozygote. To elucidate the effects of *Ptf1a* homozygous loss, we prepared *Elastase-CreERTM; Ptf1a*^*floxed*/*floxed*^ mice and found that homozygous *Ptf1a* deletion in adult acinar cells causes severe apoptosis. Electron microscopy revealed endoplasmic reticulum (ER) stress, a known cause of unfolded protein responses (UPR). We confirmed that UPR was upregulated by the activating transcription factor 6 (ATF6) and protein kinase RNA (PKR)-like endoplasmic reticulum kinase (PERK) pathways, but not the inositol requiring enzyme 1 (IRE1) pathway. Furthermore, we detected the expression of CCAAT-enhancer-binding protein (C/EBP) homologous protein (CHOP), a pro-apoptotic factor, indicating the apoptosis was induced through UPR. Our homozygous model helps clarify the role PTF1A has on the homeostasis and pathogenesis of exocrine pancreas in mice.

## Introduction

A great volume of study has identified key transcriptional factors that play central roles in cell specification, growth and differentiation during organogenesis. Even in adult cells, sets of transcriptional factors have been reported to shift cell identity to other cell types. Examples include the Yamanaka factors (*Oct3*/*4*, *Sox2*, *c-Myc* and *Klf4*) for the creation of iPS cells^1,2^, the induction of *MyoD* for the direct reprograming of fibroblasts to myoblasts^3^, and the induction of *Hnf4α* and one of *Foxa1*, *Foxa2* or *Foxa3* for the transdifferentiation to hepatic cells^4^. These studies demonstrate the dosage of key transcription factors plays an important role in the regulation of cell behavior.

*Ptf1a* is an indispensable gene for pancreas formation during organogenesis^5,6^. Using Cre-mediated lineage tracing experiments, we have previously demonstrated that PTF1A functions as a pancreas-fate determinant; the progeny of *Ptf1a*-expressing cells are committed to the pancreatic cell fate, whereas the progeny of *Ptf1a*-deficient cells are committed to the duodenal or bile duct cell fate^6^. While PTF1A is broadly expressed in nascent pancreatic buds, its expression is gradually confined to exocrine lineage during development and finally to acinar cells^5–8^. Analyses of *Ptf1a* hypomorphic mutant mice revealed that there exists a threshold of the *Ptf1a* mRNA dosage that allows pancreatic-fate specification and progression along the proper developmental pathway; a reduction of *Ptf1a* mRNA dosage resulted in a decrease in the number of cells that adopt the pancreatic cell fate, a reduction in cell proliferation of early pancreatic precursors, and an impairment of exocrine cytodifferentiation^9^.

Despite accumulating information on PTF1A function and PTF1A dosage during embryonic pancreatogenesis, knowledge on the role of PTF1A in adult pancreas is limited. Originally, PTF1A was found as a transcriptional regulator of digestive enzymes such as amylase and elastase in adult acinar cells^7^. Recently, Krah *et al*. showed that conditional deletion of *Ptf1a* in adult acinar cells resulted in ductal metaplasia and made the cells hypersensitive to *Kras* transformation^10^. In addition, Hoang *et al*. reported that *Ptf1a* deletion in adult acinar cells promotes the expression of genes consistent with stomach lineage^11^. These reports support the notion that PTF1A is required for maintaining acinar cell identity in adults. However, because the studies used *Ptf1a*^*CreER*/*floxed*^ compound heterozygote mice, the dosage effect of PTF1A remains unexplored. Considering that adult acinar cells in *Ptf1a* heterozygous mice proliferate more than wild type mice^12^ and that oncogenic *Kras*-induced pancreatic cancer progresses more rapidly in *Ptf1a* heterozygous mice^10^, the original PTF1A dosage may affect the observations made in these *Ptf1a* conditional knockout studies.

To explore the dosage effects of PTF1A on adult acinar cells, we used *Elastase-CreERTM; Ptf1a*^*floxed*/*floxed*^ mice to inactivate PTF1A and tracked the fate of *Ptf1a*-deleted cells by lineage tracing analyses. We found that *Ptf1a* deletion caused not only a shift in identity to duct cells but also severe apoptosis in acinar cells, which resulted in a rapid reduction of pancreatic mass. Furthermore, we found evidence that the changes were associated with ER stress through activation of the PERK-eIF2α-ATF4 and ATF6 pathways and induction of the pro-apoptotic factor CHOP.

## Results

### *Ptf1a* conditional knockout caused pancreatic volume loss and acinar apoptosis

We interbred *Elastase-CreERTM* and *Ptf1a*^*floxed*^ mice to obtain *Elastase-CreERTM; Ptf1a*^*floxed*/*floxed*^ mice (Ptf1a cKO mice) and *Elastase-CreERTM; Ptf1a*^+/+^ mice (control) and then crossed them with *Rosa26R* or *Rosa26-RFP* mice for lineage tracing (Supplementary Fig. S1) and injected tamoxifen (0.2 mg/g body weight) at the adult stage. We confirmed the efficiency of PTF1A depletion after Cre-mediated recombination by PTF1A immunostaining. PTF1A positivity per lineage-labeled acinar cells was 74.0 ± 6.9% in control mice and 4.4 ± 2.8% in Ptf1a cKO mice on day 3, and 84.2 ± 1.8% in control mice and 1.9 ± 0.4% in Ptf1a cKO mice on day 10, indicating satisfactory depletion of PTF1A in Ptf1a cKO mice (Supplementary Fig. S2a,b).

**Figure 1:**
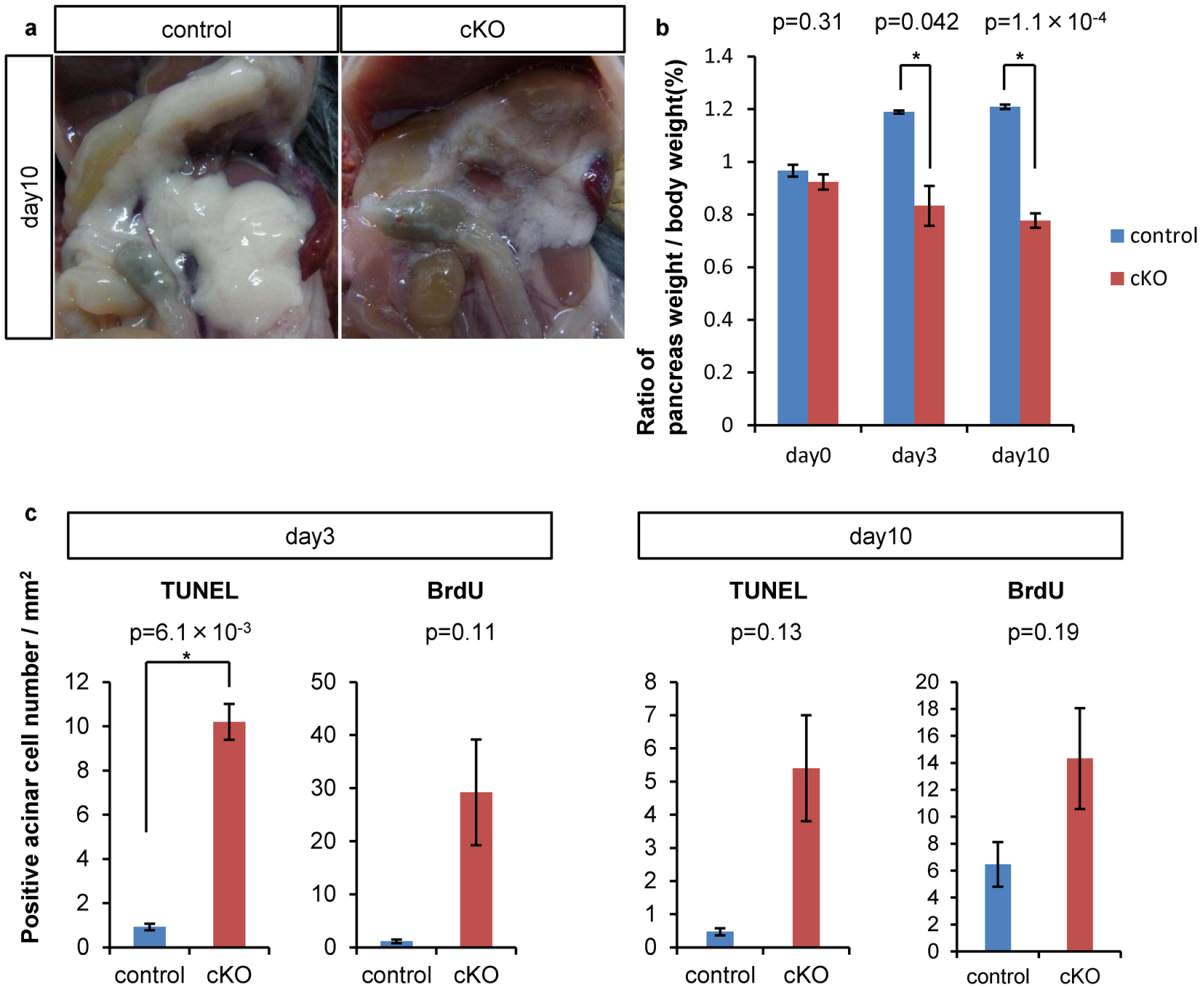
Acinar-specific *Ptf1a* deletion decreased pancreatic volume through activated apoptosis. (**a**) Representative pancreatic appearance of control and Ptf1a cKO mice on day 10. (**b**) Relative pancreatic weight per body weight. control, n = 3; Ptf1a cKO, n = 3. (**c**) The number of TUNEL or BrdU positive acinar cells per mm^2^ on day 3 or 10. control, n = 3; Ptf1a cKO, n = 3. *P < 0.05.

The pancreas of Ptf1a cKO mice was significantly edematous on day 10 (Fig. 1a), but had already reduced in size by day 3 (Fig. 1b). To account for the size reduction, we observed acinar-to-ductal metaplasia (ADM) in Ptf1a cKO mice^10,11^. The ADM area was only 2.5% and 1.5% of the whole pancreas on days 3 and 10, respectively, in Ptf1a cKO mice (Supplementary Fig. S3). Considering that the ratio of pancreas weight per body weight of Ptf1a cKO mice was about two thirds that of control mice (Fig. 1b), ADM alone could not explain the pancreatic size reduction. Indeed, TUNEL staining revealed significantly more cell death by day 3 in Ptf1a cKO mice than in control mice, but not on day 10 (Fig. 1c). On the other hand, the number of proliferative (BrdU(+)) cells between control and mutant mice was the same on day 3 and the same on day 10 (Fig. 1c). Thus, accelerated apoptotic cell death by day 3 is presumably the main cause of the pancreatic mass reduction in the mutants.

**Figure 2:**
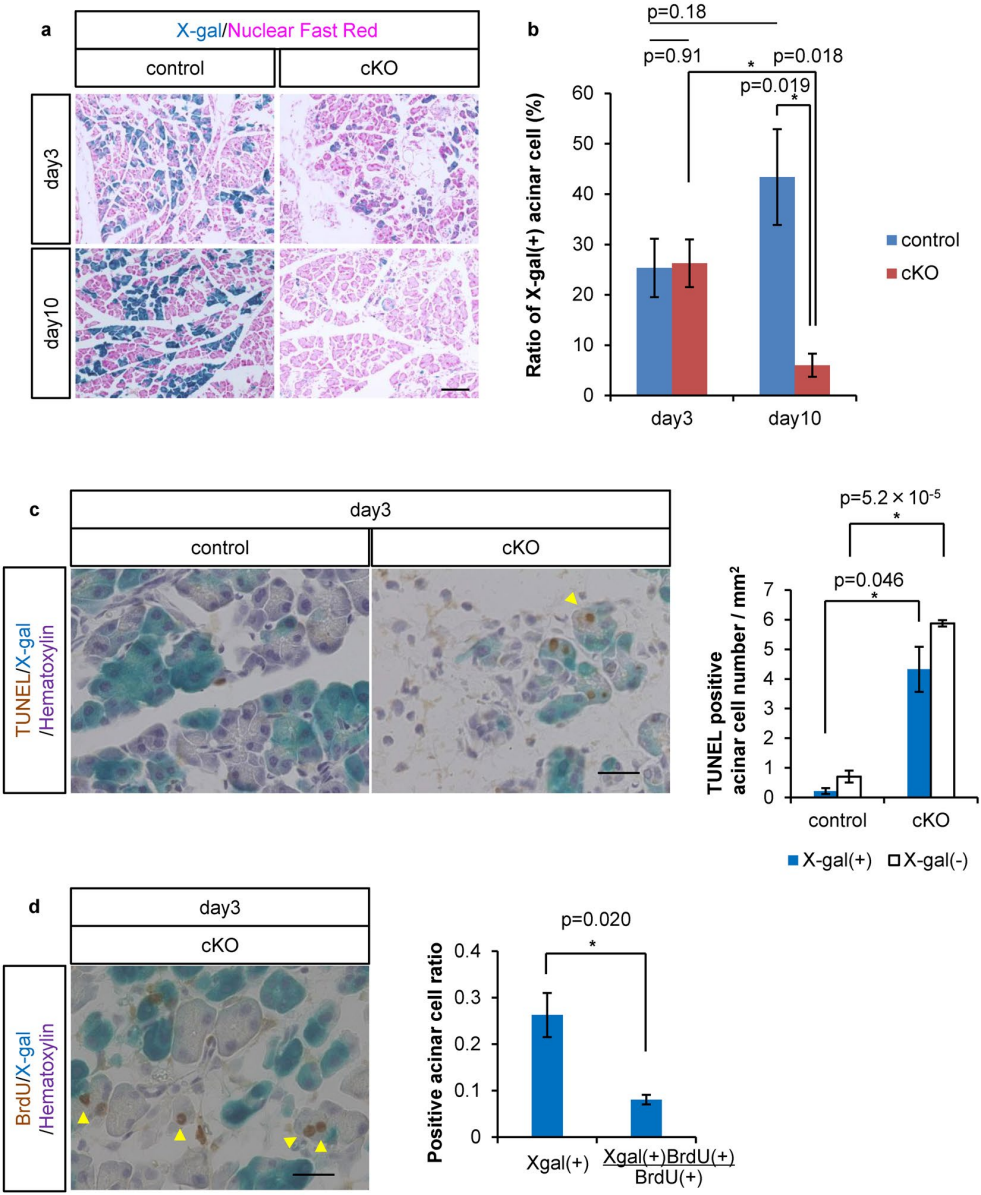
Lineage tracing revealed activated apoptosis and compensatory proliferation in Ptf1a cKO pancreata. (**a**) Representative figures of control and Ptf1a cKO mice on day 3 and day 10 stained by X-gal and Nuclear Fast Red. (**b**) The ratio of X-gal labeled acinar cells. control, n = 3; Ptf1a cKO, n = 3. (**c**) Representative figures of TUNEL staining with X-gal and hematoxylin staining, and the number of TUNEL(+) acinar cells per mm^2^ on day 3. (**d**) Representative figure of BrdU staining with X-gal and hematoxylin staining, and the ratio of X-gal(+)BrdU(+) cells per total BrdU(+) cells compared with the ratio of X-gal(+)cells in Ptf1a cKO mice on day 3. Arrowheads indicate an X-gal(−)TUNEL(+) nucleus in (**c**) or X-gal(−)BrdU(+) nuclei in (**d**) in Ptf1a cKO mice. Scale bars = 100 μm (**a**) or 25 μm (**c**,**d**). *P < 0.05.

Consistently, an accelerated cell death of PTF1A-depleted cells was supported by the reduction of lineage-labeled cells in Ptf1a cKO mice on day 10 (43.4 ± 9.5% in control vs. 6.0 ± 2.3% in Ptf1a cKO mice; see Fig. 2a,b). Interestingly, our lineage tracing analyses revealed non-autonomous cell death in the *Ptf1a*-preserved (X-gal(−)) population of Ptf1a cKO mice on day 3 (Fig. 2c). At the same time, a compensatory proliferation of surrounding *Ptf1a*-preserved acinar cells in mutant mice was suggested to have occurred by day 3. As shown in Fig. 2d, we detected BrdU(+) cells in the X-gal(−) population. If the proliferative cells were equally distributed independently of *Ptf1a* deletion, the ratio of X-gal(+) should be equivalent to the ratio of X-gal(+)BrdU(+)cells per total BrdU(+) cells. However, we found the X-gal(+) ratio was higher, suggesting that *Ptf1a*-preserved cells proliferated more than *Ptf1a*-deleted cells in mutant pancreata at this stage. Cell death and proliferation seemed to be balanced on day 3, because the percentage of Cre-recombinase activated (X-gal(+)) acinar cells was unchanged between the two mouse types (25.3 ± 5.8% in control and 26.3 ± 4.7% in Ptf1a cKO mice; see Fig. 2a,b). However, compensatory proliferation could not maintain the organ size, and the percentage of X-gal(+) acinar cells was significantly decreased on day 10 (see Figs 1a and 2a,b). Furthermore, the pancreatic volume did not recover by day 20 (Supplementary Fig. S4), which is consistent with a previous report^11^. The precise mechanism mediating the interaction between *Ptf1a*-deleted and *Ptf1a*-preserved cells warrants future investigation.

### Electron microscopy unveiled endoplasmic reticulum stress

**Figure 3:**
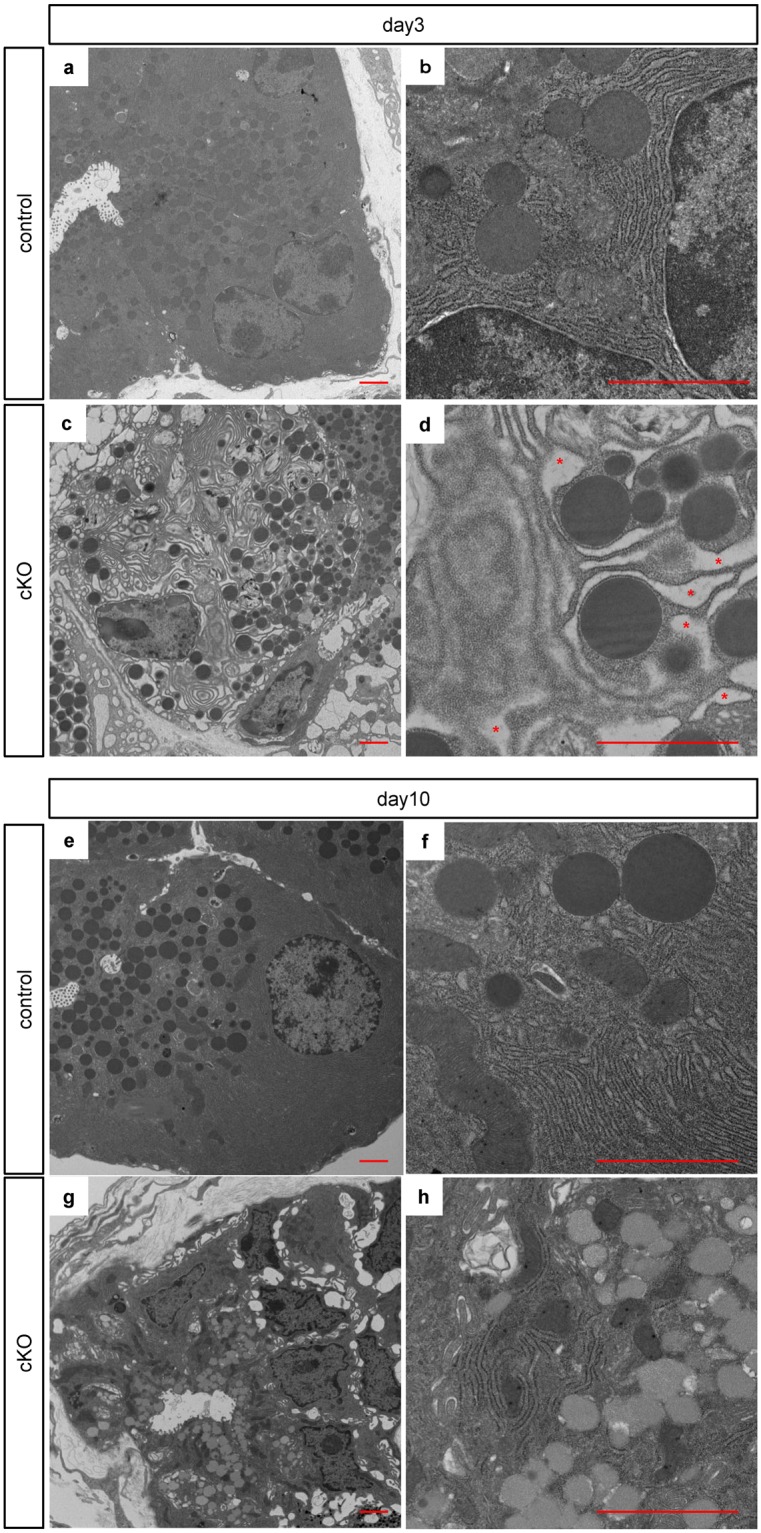
Activated endoplasmic reticulum stress in acinar cells of Ptf1a cKO mice. Representative findings of electron microscopy in control mice on day 3 (**a**) in Ptf1a cKO mice on day 3 (**c**) in control mice on day 10 (**e**) and in Ptf1a cKO mice on day 10 (**g**). (**b**,**d**,**f**,**h**) are magnified images of (**a**,**c**,**e**,**g**). Note the dilated ER lumen (indicated by red asterisks) in Ptf1a cKO mice on day 3 (**d**). Scale bars = 2 μm.

To investigate the cell death machinery in Ptf1a cKO mice, we performed electron microscopic analyses (Fig. 3). We noticed the emergence of abnormal acinar cells characterized by significantly dilated ER lumen in Ptf1a cKO pancreata on day 3 (compare Fig. 3b,d). This characteristic phenotype suggests ER stress was caused by an accumulation of unfolded or misfolded proteins within the ER lumen^13,14^. The ER lumen size was restored to normal by day 10 in mutant mice (compare Fig. 3e–h).

### ATF6 cleavage was higher in Ptf1a cKO mice on day 3

**Figure 4:**
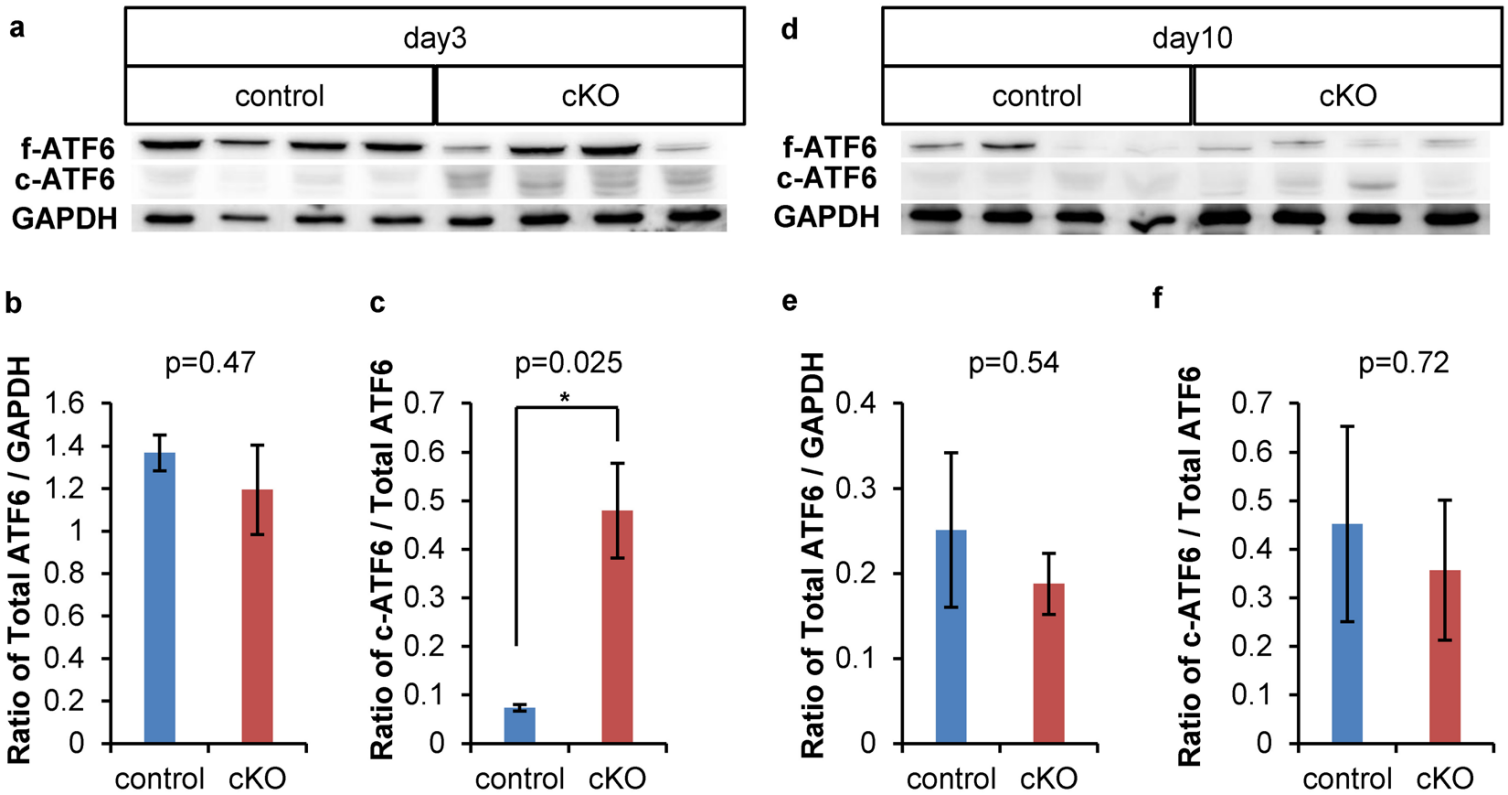
Cleavage of ATF6 was upregulated in Ptf1a cKO mice on day 3 but recovered by day 10. (**a**) Western blotting of control and Ptf1a cKO mice on day 3. (**b**) Comparison of the ratio of total ATF6/GAPDH expression in control and Ptf1a cKO mice on day 3. (**c**) Comparison of the ratio of cleaved ATF6/total ATF6 expression in control and Ptf1a cKO mice on day 3. (**d**) Western blotting of control and Ptf1a cKO mice on day 10. (**e**) Comparison of the ratio of total ATF6/GAPDH expression in control and Ptf1a cKO mice on day 10. (**f**) Comparison of the ratio of cleaved ATF6/total ATF6 expression in control and Ptf1a cKO mice on day 10. f-ATF6: full length ATF6, c-ATF6: cleaved ATF6. control, n = 4; Ptf1a cKO, n = 4. *P < 0.05. The original, unprocessed scans of the blots are shown in Supplementary Fig. S5.

Our electron microscopy observations prompted us to analyze the unfolded protein response (UPR) to ER stress including activation of the ATF6, IRE1 and PERK-eIF2α-ATF4 pathways, which can contribute to apoptosis^15^. Western blotting revealed that total ATF6 expression was similar in the two mouse groups on days 3 and 10 (Fig. 4a,b,d,e). However, cleaved ATF6 was more highly expressed in Ptf1a cKO mice on day 3 but not on day 10 (Fig. 4a,c,d,f), indicating that the ATF6 pathway was activated on day 3 but deactivated by day 10 in Ptf1a cKO mice.

### *Xbp1* mRNA splicing was suppressed in Ptf1a cKO mice on day 3

**Figure 5:**
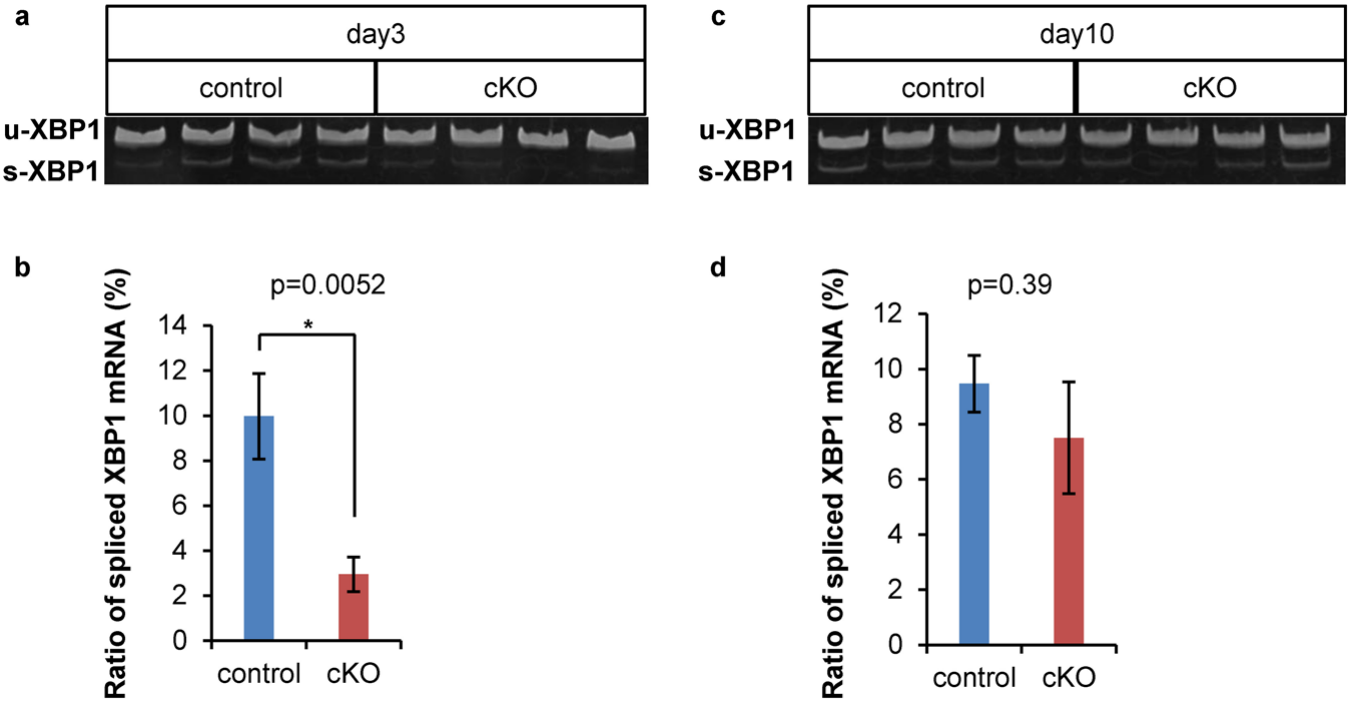
Splicing of *Xbp1* mRNA was suppressed in Ptf1a cKO mice on day 3 but recovered by day 10. (**a**) Representative electrophoresis of RT-PCR products of unspliced and spliced *Xbp1* mRNA on day 3. (**b**) Comparison of the percentage of spliced *Xbp1* mRNA on day 3 in control and Ptf1a cKO mice. control, n = 5; Ptf1a cKO, n = 6. (**c**) Representative electrophoresis of RT-PCR products of unspliced and spliced *Xbp1* mRNA on day 10. (**d**) Comparison of the percentage of spliced *Xbp1* mRNA on day 10 in control and Ptf1a cKO mice. control, n = 6; Ptf1a cKO, n = 5. *P < 0.05. The original, unprocessed scans of the gels are shown in Supplementary Fig. S6.

Next, we investigated the IRE1 pathway, which splices X-box binding protein 1 (*XBP1*) mRNA in response to ER stress in mammals^16^ (Fig. 5). Our RT-PCR analyses revealed a lower ratio of spliced *Xbp1* mRNA in Ptf1a cKO mice on day 3 but not on day 10, suggesting that the IRE1 pathway was suppressed on day 3 but recovered by day 10 in Ptf1a cKO mice.

### ATF4 and CHOP were activated in Ptf1a cKO mice on day 3

**Figure 6:**
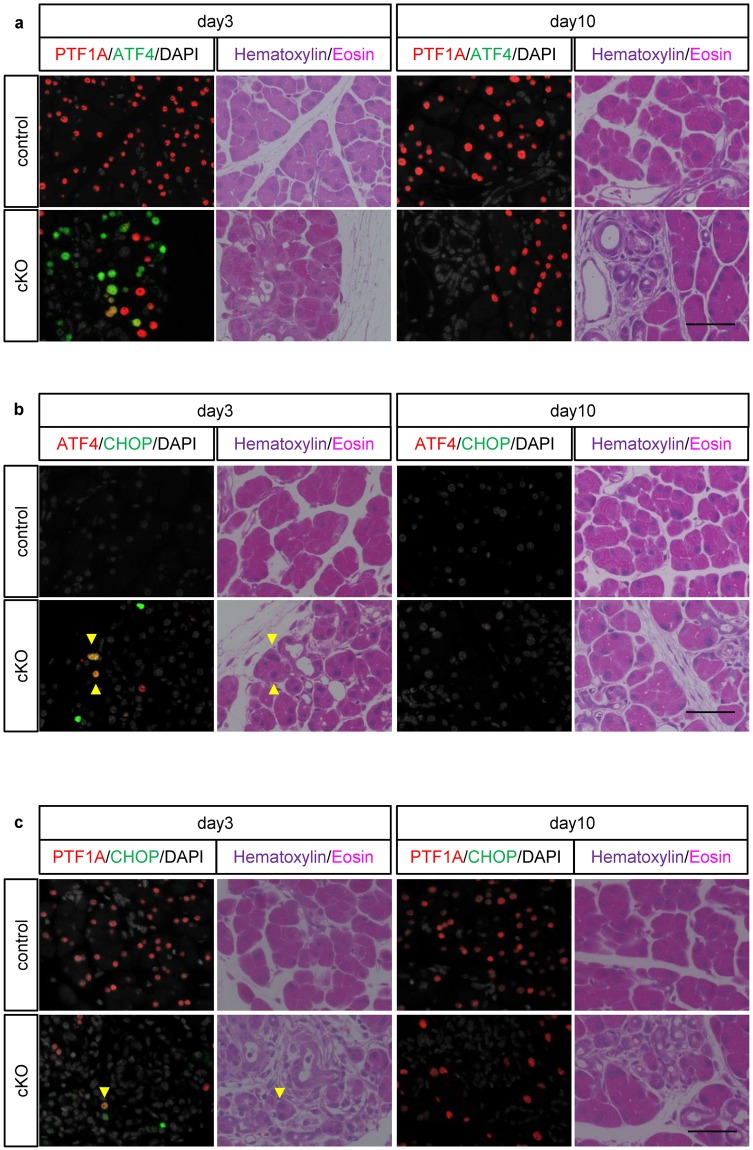
ATF4 and CHOP were upregulated in Ptf1a cKO mice on day 3 but recovered by day 10. Immunofluorescence of (**a**) PTF1A and ATF4 (**b**) ATF4 and CHOP, and (**c**) PTF1A and CHOP with hematoxylin-eosin staining on days 3 and 10. Arrowheads indicate CHOP and ATF4 double positive nuclei in (**b**) or a PTF1A and CHOP double positive nucleus in (**c**). Hematoxylin-eosin was stained on the same sections after immunofluorescence. Scale bars = 50 μm.

Finally, we examined the PERK-eIF2α-ATF4 pathway by ATF4 immunostaining. In control mice, no ATF4 signal in acinar cells was observed. On the other hand, in Ptf1a cKO mice, there was sporadic ATF4 expression predominantly in PTF1A-absent cells on day 3 and almost no ATF4 expression on day 10 (Fig. 6a). Thus, both the ATF6 pathway (Fig. 4) and the PERK-eIF2α-ATF4 axis were upregulated on day 3 but returned to normal activation status by day 10 in Ptf1a cKO mice, which is consistent with our electron microscopy observations (Fig. 3). Furthermore, some cells on day 3 showed immunoreactivity for CHOP, a pro-apoptotic factor^17,18^, but not on day 10 (Fig. 6b). CHOP immunoreactivity was observed in both ATF4(+) and ATF4(−) cells. Considering that CHOP is a downstream target of the ATF6 and PERK-eIF2α-ATF4 pathways^19,20^, ATF4(−)CHOP(+) cells may represent activation of the ATF6 pathway. Taken together, these findings suggest that acinar cell death in Ptf1a cKO mice is caused by excessive UPR. Intriguingly, we observed rare CHOP-PTF1A double positive cells in the mutants on day 3 (Fig. 6c), suggesting non-autonomous cell death in *Ptf1a*-preserved cells (Fig. 2c).

## Discussion

Here we show that conditional *Ptf1a* loss in adult acinar cells causes ER stress and activates apoptosis pathways to decrease pancreatic size. It is known that the accumulation of unfolded or misfolded proteins in the ER causes ER stress^13,14^. In response, cells escape from the stress status by activating the PERK-eIF2α-ATF4, ATF6 and IRE1 pathways^15^. Each of these three pathways has a specific effect on the cell response to ER stress. PERK phosphorylates eIF2α^21^ to attenuate the translation of most genes except those that have a specific upper open reading frame such as ATF4^22^, thereby preventing additional stress acutely^15^. Cleaved ATF6 activates an ER chaperone to restore the protein folding machinery^20,23^. IRE1 splices *Xbp1* mRNA to promote protein degradation^24^ and thus decrease ER stress^15^. These mechanisms protect the cell from ER stress, but under excessive stress intensity and duration, prolonged expression of ATF4 and cleaved ATF6 upregulates the pro-apoptotic protein CHOP, which promotes cell death^15,17–20^. In Ptf1a cKO mice, we confirmed activation of the PERK-eIF2α-ATF4 and ATF6 pathways plus subsequent CHOP upregulation, and suppression of the IRE1 pathway. These findings suggest acinar cells in mutant mice are not in a cytoprotective state.

Unlike in our model, Krah *et al*. showed no difference in acinar cell death between control and Ptf1a cKO mice on day 14^10^, while Hoang *et al*. observed more apoptosis and UPR change in Ptf1a cKO mice than control^11^. In addition, consistent with the autophagy they detected, they showed that *Xbp1* mRNA splicing was upregulated on days 6 and 14, indicating a cytoprotective response. On the other hand, consistent with the ER stress we detected, we showed different UPR on day 3, indicating an apoptotic response. We speculate that the phenotypic differences between ours and previous models might be explained by the original PTF1A dosage before the depletion. In our experiments, PTF1A dosage decreased from the homozygous-to-null status, whereas in previous models the dosage decreased from the heterozygous-to-null status. Therefore, a more dynamic change in PTF1A expression should have occurred in our model. Presumably, adult acinar cells could not adapt to the rapid and massive decrease of PTF1A expression that caused severe ER stress.

At the same time, several observations were consistent with ours and previous reports. For example, we observed ADM in Ptf1a cKO mice. ADM cells may have escaped the apoptotic cell death machinery and survived by discarding their cell identity as acinar cells. Krah *et al*. provided evidence that CD45(+) immune cells accumulate upon *Ptf1a* deletion and that caerulein-induced pancreatitis causes more ADM in Ptf1a cKO mice^10^. Considering that caerulein-induced pancreatitis itself reduces PTF1A expression^25^ and causes ER stress^26^ in acute phase, we propose there exists an interdependence among inflammation, ER stress and the maintenance of acinar cell identity or cell death, in which PTF1A expression plays a crucial role. In this study, we observed accelerated apoptosis and compensatory proliferation of the *Ptf1a*-preserved cells in the mutant pancreata. These observations indicate the competition between *Ptf1a*-deleted and -preserved cells, in which non-cell autonomous regulation plays a role. The precise mechanism including the identification of responsible signals warrants future investigation.

In summary, we demonstrated that the homozygous-to-null reduction of *Ptf1a* triggered excessive UPR through activation of the ATF6 and PERK-eIF2α-ATF4 pathways and suppression of the IRE1 pathway in adult acinar cells. Future studies are required to dissect how acinar cells initiate apoptosis or survive (for example, by changing their identity from acinar to duct-like) upon the rapid and dynamic reduction of PTF1A expression.

## Methods

### Mice

*Elastase-CreERTM*, *Rosa26R*, *Rosa26-RFP* and *Ptf1a*^*floxed*^ mice were previously described^27–30^. By mating, we obtained *Elastase-CreERTM; Ptf1a*^*floxed*/*floxed*^*; Rosa26R* or *Rosa26-RFP* mice (Ptf1a cKO mice) in which *Ptf1a*-deleted cells are detected as X-gal(+) or RFP(+) cells after tamoxifen injection. All animal experiments were performed in accordance with the Kyoto University guidelines for animal experiments and approved by the animal research committee of Kyoto University.

### Drug injection

For lineage tracing, 8-week-old mice were injected once intraperitoneally with tamoxifen (T5648, Sigma-Aldrich, St. Louis, MO, USA) at 0.2 mg/g body weight. For BrdU staining, mice were injected intraperitoneally with BrdU (Merck Millipore, Billerica, MA, USA) at 2 mg 1 hour before euthanization.

### Tissue preparation

All tissue preparations were performed as previously described^31^ with some modification. Briefly, for X-gal staining, ice-cold fixative solution (4% paraformaldehyde (PFA), 1% glutaraldehyde (GA)/PBS) was perfused into mice, and specimens were immersed into 4% PFA/PBS for 2 hours at 4 °C followed by 30% sucrose/PBS until equilibration and then embedded into O.C.T. compound (Sakura, Osaka, Japan). For raw RFP sections, the fixative solution perfused was 4% PFA/PBS, and specimens were immersed into the same solution for 4 hours at 4 °C followed by 30% sucrose/PBS until equilibration and then embedded into O.C.T. compound. For paraffin sections, the fixative solution perfused was 4% PFA/PBS, and specimens were immersed into the same solution overnight at 4 °C, then dehydrated in graded alcohol, immersed in Histo-Clear (National Diagnostics, Atlanta, GA, USA) and finally embedded into paraffin.

### X-gal staining, immunohistochemistry, immunofluorescence and alcian blue staining

Frozen sections were cut into 4-μm-thick slices. For X-gal staining, they were reacted at room temperature overnight as previously reported^31^ with some modifications. For TUNEL assays, DeadEnd Colorimetric Apoptosis Detection System (Promega Corporation, Madison, WI, USA) was used in accordance with the manufacturer’s protocols. Paraffin sections were cut into 2-μm-thick slices, deparaffinized and rehydrated. For either immunohistochemistry or immunofluorescence, the procedures from antigen retrieval to antibody reaction were done as previously reported^32^, however, heat induced epitope retrieval was omitted in the cases of raw RFP detection. The primary antibodies used are listed in Supplementary Table S1 and the secondary antibodies in Supplementary Table S2. The slices were treated with DAB Peroxidase Substrate (SK-4100, Vector Laboratories) for immunohistochemistry or with TSA Plus System (PerkinElmer, Waltham, MA, USA) and 4,6-diamidino-2-phenylindole (DAPI) for immunofluorescence. For alcian blue staining, sections were immersed into 3% acetic acid and then reacted with Alcian Blue Stain Solution pH 2.5 (Muto Pure Chemicals, Tokyo, Japan) for 20 minutes. Sections were counterstained by Nuclear Fast Red (Seracare, Milford, MA, USA), hematoxylin and/or eosin (Wako, Osaka, Japan), as previously reported^31^. Images were visualized using HS All-in-one Fluorescence Microscopy BZ-9000E (Keyence, Osaka, Japan).

### Cell counting

For labeled cell assessment, we chose 30 fields (3 random views per section, 10 sections at equal intervals) at ×200 magnification, counted X-gal positive and negative acinar cells (at least 20,000 cells) in each mouse and calculated the ratio. For PTF1A depletion assessment, we counted at least 700 RFP(+) cells from 30 or 50 fields (3 or 5 random views per section, 10 sections at equal intervals) in each mouse. For BrdU and TUNEL analyses, positive cells were counted in 50 fields (5 random views per section, 10 sections at equal intervals).

### Alcian blue(+) area evaluation

10 paraffin sections at equal intervals were stained by alcian blue, and whole images were taken. Whole pancreatic and alcian blue(+) areas were enclosed manually and quantified with BZ-H1M software (Keyence).

### Electron microscopy

For tissue preparation, 4% PFA and 2% GA/PBS was perfused into mice. Specimens were cut into approximately 1 mm^3^ sections, immersed into the same solution overnight at 4 °C, and brought to Division of Electron Microscopic Study (DEMS), Center for Anatomical Studies, Graduate School of Medicine, Kyoto University. DEMS conducted the osmium tetroxide postfixation and the preparation of ultrathin sections with uranium staining. The sections were observed using a transmission electron microscope (Hitachi H-7650, Hitachi, Tokyo, Japan).

### Western blotting

Whole pancreatic tissues were used. The procedures from lysate preparation to antibody reaction were reported previously^32^. The primary antibodies used are listed in Supplementary Table S3 and the secondary antibodies in Supplementary Table S4. Chemiluminescence was detected with Chemi-Lumi One Super (Nacalai tesque, Kyoto, Japan) and visualized with ImageQuant LAS 4000 (GE Healthcare, Chicago, IL, USA). The intensity of the bands was quantified with ImageQuant TL software (GE Healthcare), and the intensity ratio was calculated.

### RNA isolation and data analysis

Total pancreatic RNA was extracted using RNeasy Mini Kit (Qiagen, Hilden, Germany) in accordance with the manufacturer’s protocols. First strand cDNA synthesis was performed using ReverTra Ace qPCR RT Master Mix (Toyobo, Osaka, Japan). Measurement of the ratio of spliced to total *Xbp1* mRNA was performed by RT-PCR using forward primer 5′–AGTTAAGAACACGCTTGGGAAT–3′ and reverse primer 5′–AAGATGTTCTGGGGAGGTGAC–3′. PCR products were 172 bp for unspliced *Xbp1* mRNA and 146 bp for spliced *Xbp1* mRNA. The products were electrophoresed in 10% acrylamide gel in TBE, stained with ethidium bromide, and visualized with ChemiDoc XRS + system (Bio-Rad Laboratories, Hercules, CA, USA). The intensity of the bands was quantified with Image Lab Software (Bio-Rad Laboratories), and the intensity ratio was calculated.

### Statistical analysis

All values are shown as means ± s.e.m. All error bars represent s.e.m. All indices were analyzed using an unpaired t test (two-tailed). P < 0.05 was considered significant.

## Electronic supplementary material

supplementary information
