# Supplementary material for: Ptf1a inactivation in adult pancreatic acinar cells causes apoptosis through activation of the endoplasmic reticulum stress pathway
Source: Sci Rep. 2018 Oct 25;8:15812. doi: 10.1038/s41598-018-34093-4 (PMC6202406; doi:10.1038/s41598-018-34093-4)
Supplement: Supplementary file 1 — supplementary information [file 41598_2018_34093_MOESM1_ESM.pdf]

Supplementary Information

Ptf1a inactivation in adult pancreatic acinar cells causes apoptosis through activation of the endoplasmic reticulum stress pathway

Morito Sakikubo, Kenichiro Furuyama, Masashi Horiguchi, Shinichi Hosokawa, Yoshiki Aoyama, Kunihiko Tsuboi, Toshihiko Goto, Koji Hirata, Toshihiko Masui, Yuval Dor, Tomoyuki Fujiyama, Mikio Hoshino, Shinji Uemoto and Yoshiya Kawaguchi

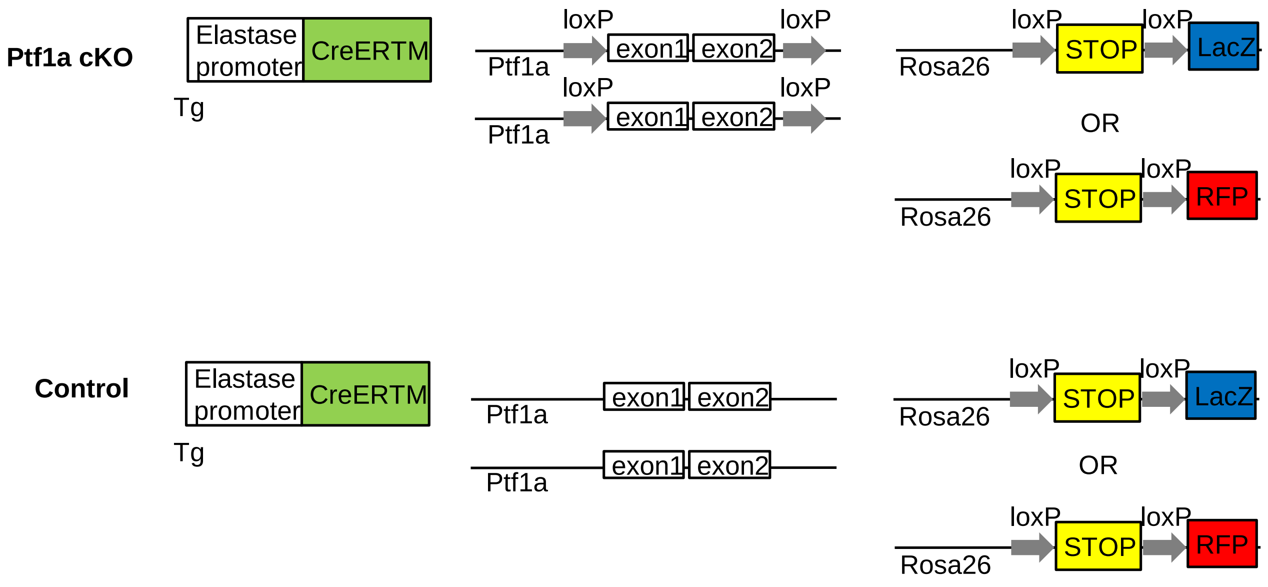

Supplementary Figure S1. Scheme of mouse breeding.
For lineage tracing, we chose *Rosa26-LacZ* (*Rosa26R*) or *Rosa26-RFP* mice.

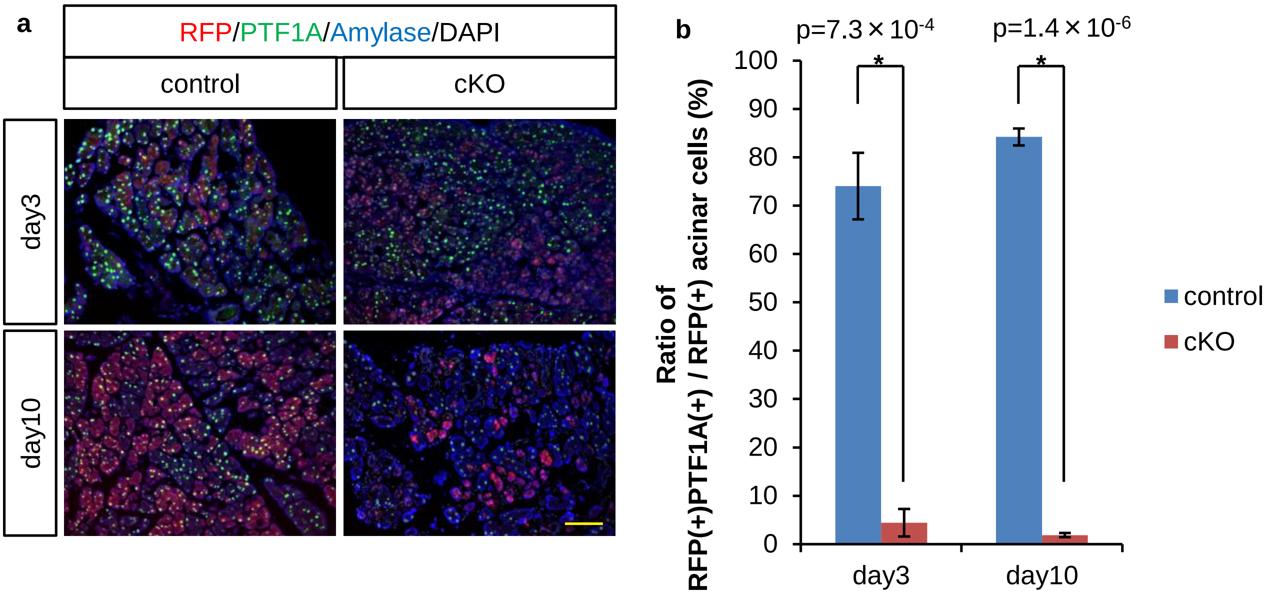

Supplementary Figure S2. Evaluation of *Ptf1a*-deleted acinar cells.
(a) Representative figures of control and Ptf1a cKO mice on days 3 and 10 stained by PTF1A, Amylase and DAPI with RFP fluorescence. (b) The ratio of RFP(+)PTF1A(+) / RFP(+) acinar cells. control, n = 3; Ptf1a cKO, n = 3. Scale bar = 100 µm. *P<0.05.

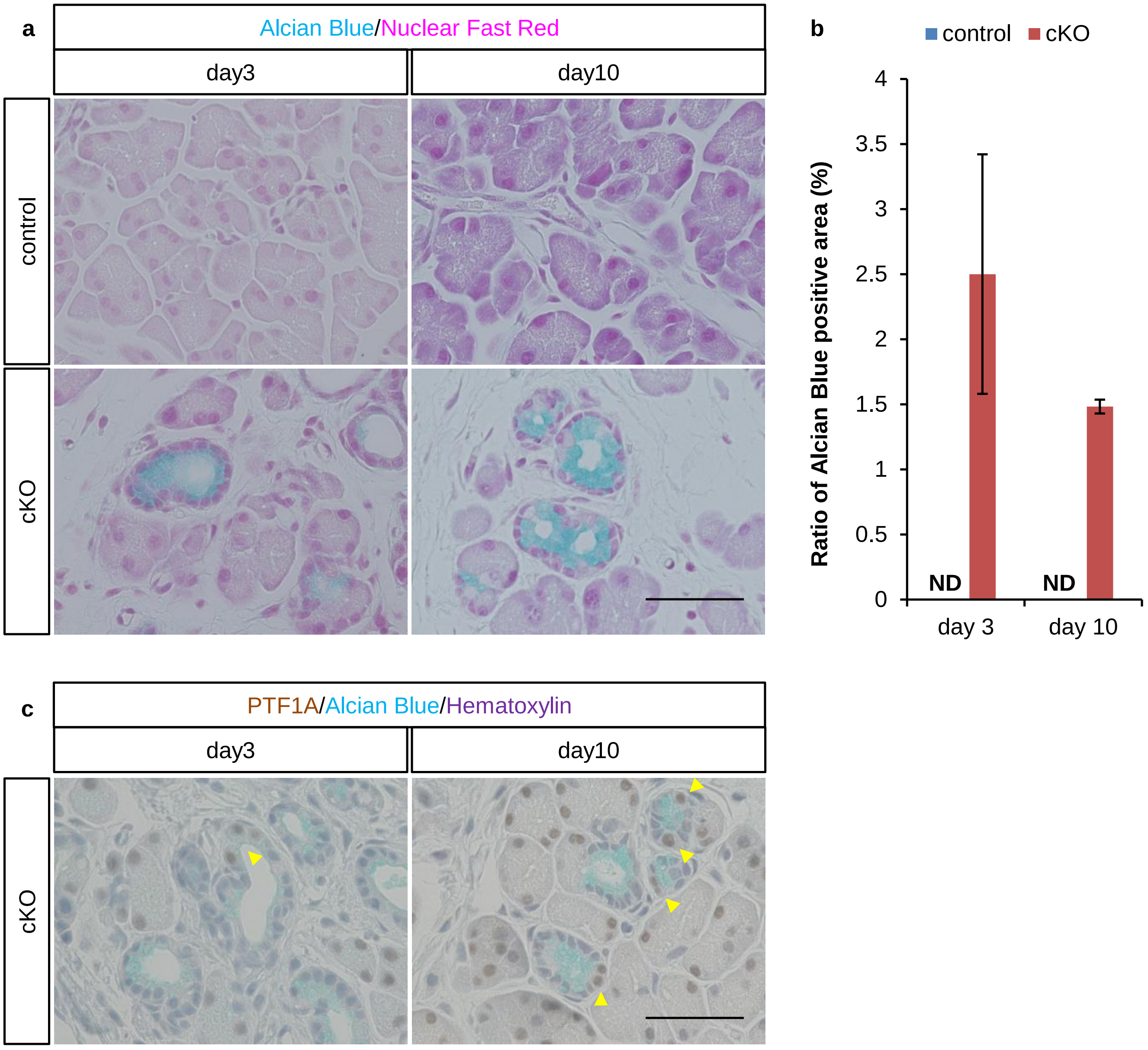

Supplementary Figure S3. Acinar-to-ductal metaplasia (ADM) in Ptf1a cKO mice.
(a) Representative figures of control and Ptf1a cKO mice on days 3 and 10 stained by alcian blue and Nuclear Fast Red. (b) Ratio of alcian blue positive area per pancreas. ADM were detected in Ptf1a cKO mice but not in control mice. control, n = 3; Ptf1a cKO, n = 3. (c) PTF1A positive cells (arrowheads) were rare in the alcian blue positive cells in Ptf1a cKO mice. We found 9 fields with PTF1A positive cells from a total of 2,381 fields counted in Ptf1a cKO mice on day 3 (n = 3) and 3 fields from a total of 2,758 fields in Ptf1a cKO mice on day 10 (n = 3). Scale bars = 50 µm.

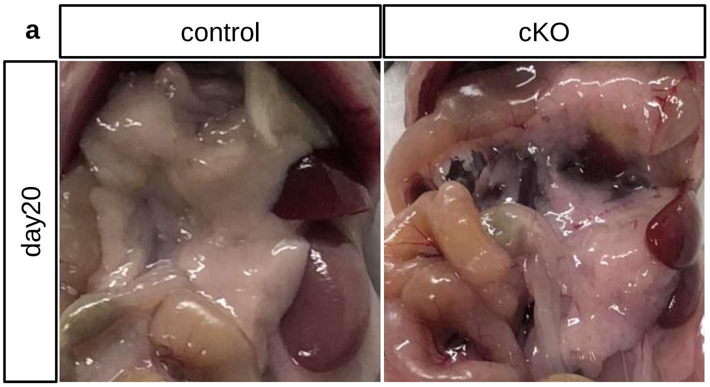

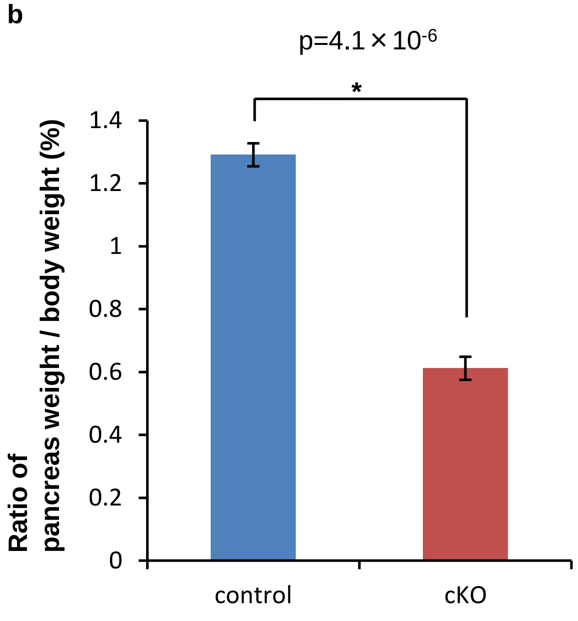

Supplementary Figure S4. Pancreas size did not recover in Ptf1a cKO mice on day 20.
(a) Representative figures of control and Ptf1a cKO mice on day 20. (b) Relative pancreatic weight per body weight. control, n = 5; Ptf1a cKO, n = 4.

For Figure 4a

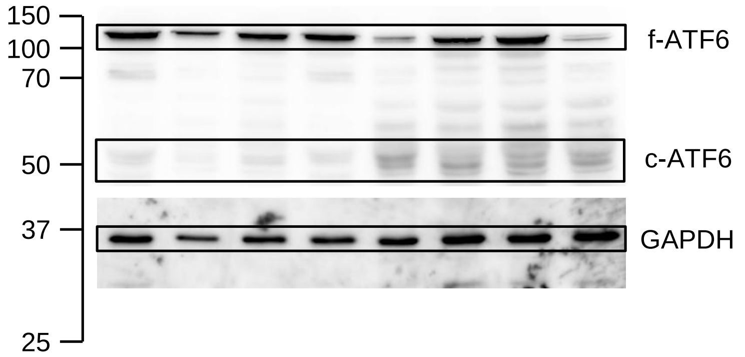

For Figure 4d

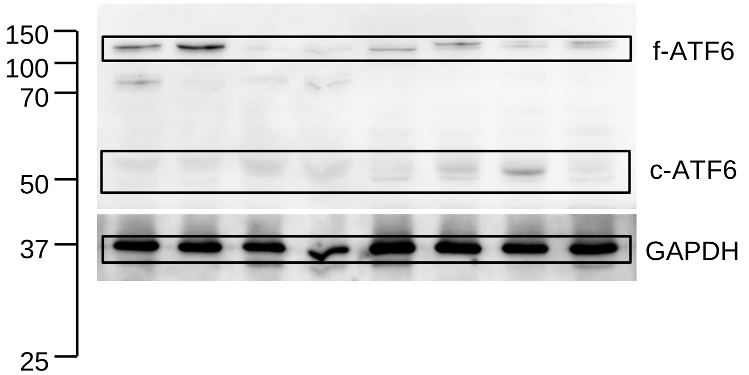

Supplementary Figure S5. Scaned images of Western blotting.
Approximate regions used for figures are marked with rectangles, and molecular sizes are indicated in kDa (based on prestained protein markers).

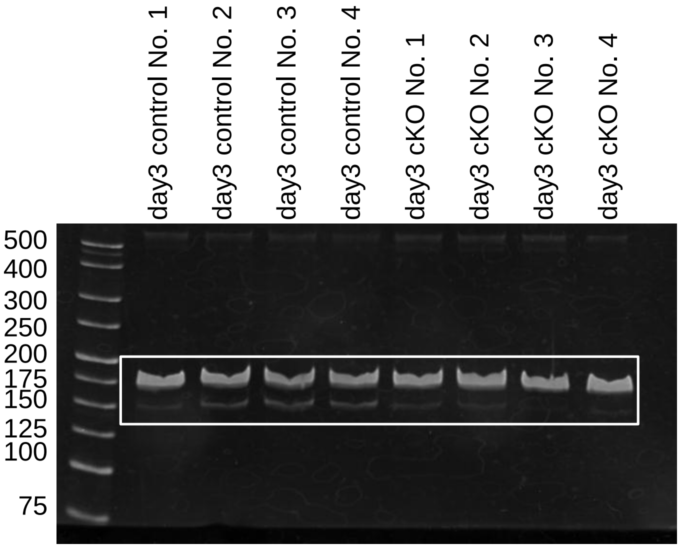

For Figure 5a

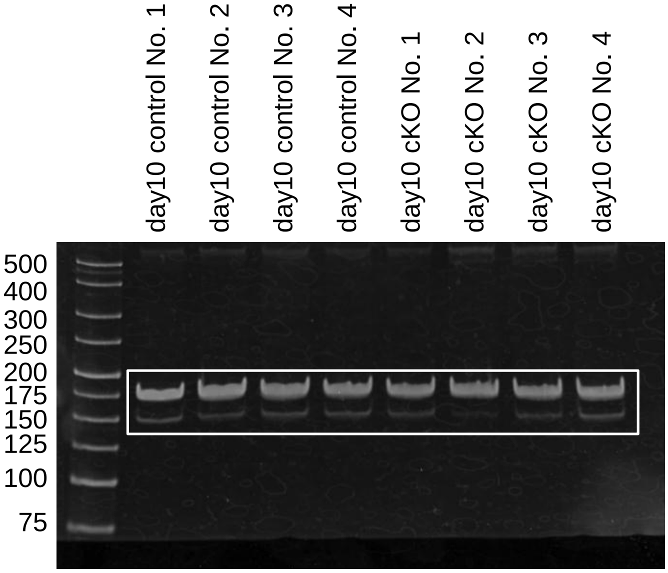

For Figure 5c

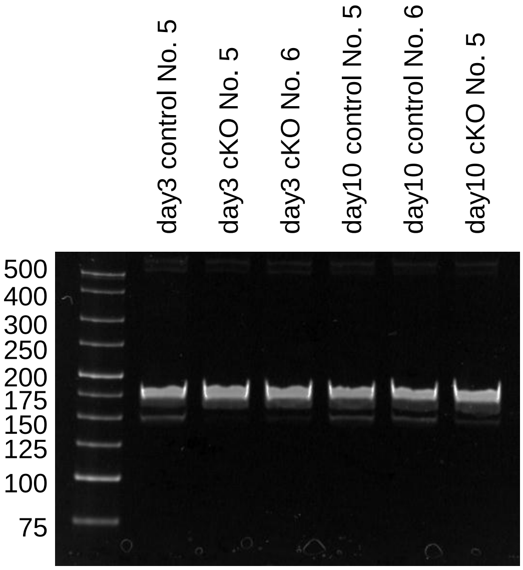

Supplementary Figure S6. Scaned images of electrophoresis gels.
Approximate regions used for figures are marked with rectangles, and molecular sizes are indicated in base pairs.

Primary antibodies	Species	Dilution	Suppliers & Codes
BrdU	Mouse	1:200	Dako<br>M0744
Amylase	Rabbit	1:1000	Sigma<br>A8273
Ptf1a	Rabbit	1:5000(frozen)<br>1:10000(paraffin)	M. Hoshino<br>NCNP
ATF4	Rabbit	1:500	CST<br>11815
CHOP	Mouse	1:5000	CST<br>2895

Supplementary Table S1.
Primary antibodies used in immunohistochemistry and/or immunofluorescence.

Secondary antibodies	Species	Dilution	Suppliers & Codes
Mouse IgG	Horse<br>HRP linked	Ready to use	Vector Laboratories<br>MP-7422
Rabbit IgG	Horse<br>HRP linked	Ready to use	Vector Laboratories<br>MP-7401

Supplementary Table S2.
Secondary antibodies used in immunohistochemistry and/or immunofluorescence.

Primary antibodies	Species	Dilution	Suppliers & Codes
ATF6	Mouse	1:200	SantaCruz<br>sc-166659
GAPDH	Rabbit	1:1000	SantaCruz<br>sc-25778

Supplementary Table S3. Primary antibodies used in western blotting.

Secondary antibodies	Species	Dilution	Suppliers & Codes
Mouse IgG	Sheep<br>HRP linked	1:5000	GE<br>NA9310
Rabbit IgG	Donkey<br>HRP linked	1:5000	GE<br>NA9340

Supplementary Table S4. Secondary antibodies used in western blotting.
